# Microfluidic cell sorter sample preparation for genomic assays

**DOI:** 10.1063/5.0092358

**Published:** 2022-06-10

**Authors:** Nicole Jagnandan, Jose Morachis

**Affiliations:** Applications, NanoCellect Biomedical Inc., San Diego, California 92121, USA

## Abstract

Single-cell RNA-Sequencing has led to many novel discoveries such as the detection of rare cell populations, microbial populations, and cancer mutations. The quality of single-cell transcriptomics relies heavily on sample preparation and cell sorting techniques that best preserve RNA quality while removing dead cells or debris prior to cDNA generation and library preparation. Magnetic bead cell enrichment is a simple process of cleaning up a sample but can only separate on a single-criterion. Droplet-based cell sorters, on the other hand, allows for higher purity of sorted cells gated on several fluorescent and scatter properties. The downside of traditional droplet-based sorters is their operational complexity, accessibility, and potential stress on cells due to their high-pressure pumps. The WOLF^®^ Cell Sorter, and WOLF G2^®^, developed by NanoCellect Biomedical, are novel microfluidic-based cell sorters that use gentle sorting technology compatible with several RNA-sequencing platforms. The experiments highlighted here demonstrate how microfluidic sorting can be successfully used to remove debris and unwanted cells prior to genomic sample preparation resulting in more data per cell and improved library complexity.

## INTRODUCTION

Single-cell transcriptomics has been used to understand the complex nature of tumor cells and specific biomarkers that correlate with disease progression and therapeutic response, including chemotherapeutic resistance.^1^ To generate accurate, high-quality data, well-suited sample preparation methods need to be used. Debris and dead cells in sequencing data can increase background noise and confound data interpretation.^2^ Sample preparation for single-cell transcriptomics can be challenging due to mechanical and physical stresses during the sample preparation process. One of the most common cell isolation methods used upstream for omics applications includes fluorescence activated cell sorters (FACS).^3^ FACS can be used to collect specific cell populations while simultaneously removing dead or dying cells and debris; therefore, maximizing the data generated per dollar spent on sequencing reagents and analysis time. However, conventional cell sorting methods that use high-pressure droplet sorting can alter cells and induce unwanted oxidative stress and metabolic pathway changes.^4,5^ Low shear-stress during cell sorting can help avoid potential stress responses induced by traditional sorters. In addition, traditional cell sorters that require the use of a specific sheath composition have been shown to contribute to sorter induced cellular stress (SICS).^6,7^ The use of microfluidic technology has grown tremendously in the last decade due to its ability to generate precise and gentle manipulation of cells. NanoCellect's WOLF^®^ Cell Sorter uses a unique disposable microfluidic cartridge that provides three important benefits: (1) elimination of sample contamination between runs, (2) sorting cells with high purity, and (3) low pressure sorting to improve cell viability. By combining the multiparameter accuracy of fluorescent antibody selection and gentleness of microfluidics, this provides an ideal setting for generating a clean sample for genomic experiments. In this paper, we demonstrate how adding this microfluidic cell sorter to omics workflows can improve sequencing results.

## MATERIALS AND METHODS

### Microfluidic sorting technology

**FIG. 1.**
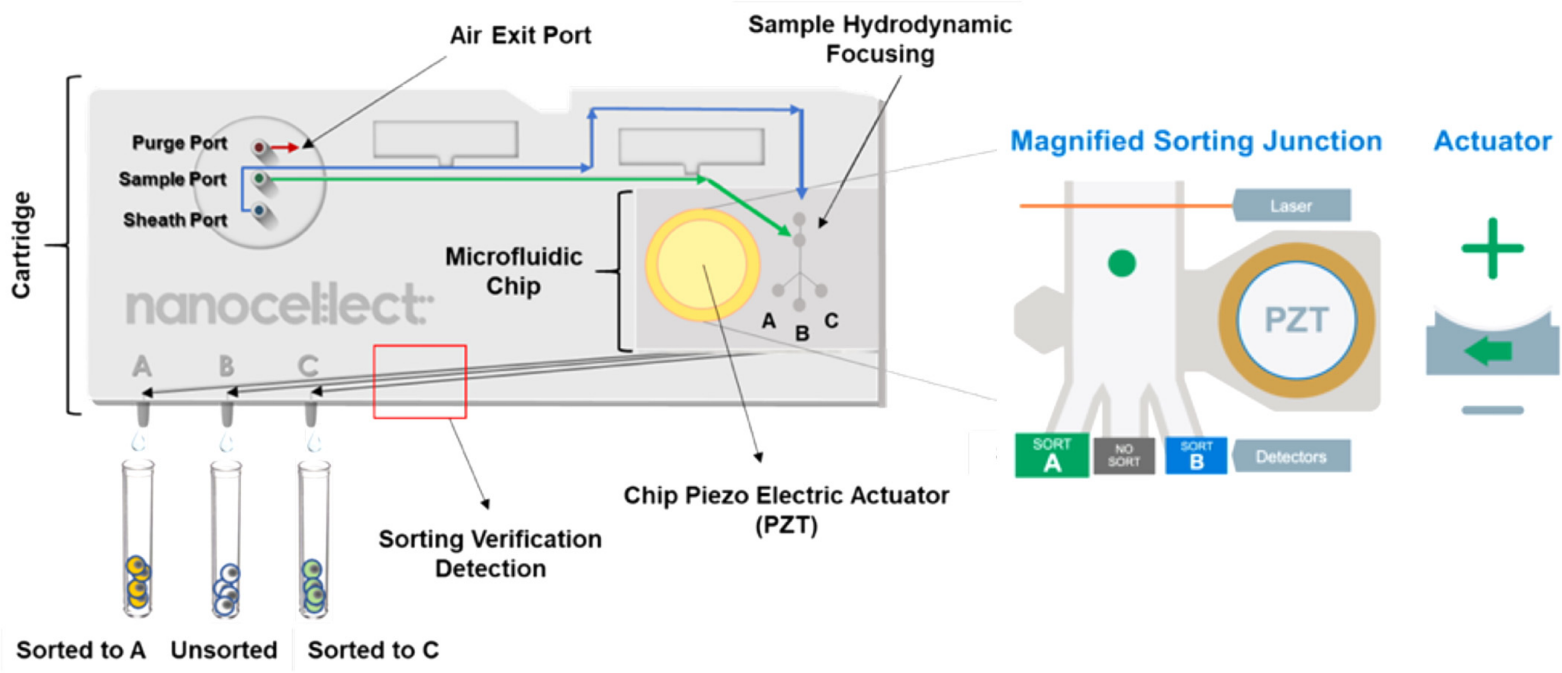
NanoCellect microfluidic sorting technology. The entire sorting process occurs on a disposable microfluidic cartridge. As the cell passes through the sorting junction, the PZT will bend to the right or left, pushing the fluid flow with the cell inside to either the left or right channels. Cells that are not of interest pass through the middle channel.

The WOLF's distinguishing capabilities are based on a microfluidic cartridge sorting technology that gently sorts cells accurately. Generating only 1.5 psi, cells are sorted within a microfluidic chip using on-chip piezoelectric actuator that deflects inward or outward mechanically, deflecting the target cell within the fluid flow into the designated sorting channel.^8–10^ Unlike the traditional electrostatic droplet sorting method, no direct forces or charges are applied to cells. The piezoelectric actuator deflection only causes the fluid stream that contains the cell to shift direction into the appropriate sorting channel, which further lowers shear stress on cells. In addition, cells are sorted at 200 cells/s at a fixed flow rate of 24 *μ*l/min for the sample and 160 *μ*l/min for sheath, this volume exits three output channels that facilitates the option to sort two targeted populations at the same time (channels A and C); while the negative populations flow through the center channel (channel B) (Fig. 1). The WOLF's microfluidic cartridges are completely disposable; everything the sample and sheath fluid touches is sterile and free from sample-to-sample contamination enabling more accurate sequencing results. In addition, sheath fluid can be anything the user believes to be most optimal for their cell type which further lowers the chance of cellular stress as demonstrated by Munoz *et al.* when sorting various cell lines on the WOLF.^11^ The WOLF Cell Sorter was used to sort peripheral blood mononuclear cells (PBMCs) and induced pluripotent stem cells (iPSCs) to demonstrate the genomic benefits that are enabled with this gentle sorting technology. The WOLF uses a single 488 nm laser to illuminate samples and collect label-free forward scatter (FWD) and backscatter (BSC) to measure the size and internal complexity of cells, respectively. This same laser can excite fluorescent emissions that can be detected in the green (510–550 nm), yellow (565–605 nm), and red (665+ nm) wavelengths. For more complex cell types requiring more fluorescent antibody markers, the WOLF G2 has 2 lasers available in three different laser configurations which include the 488 nm laser with one of the following 405 nm, 561 nm, or 637 nm lasers.

### Single-cell RNA sequencing of PBMCs

**FIG. 2.**
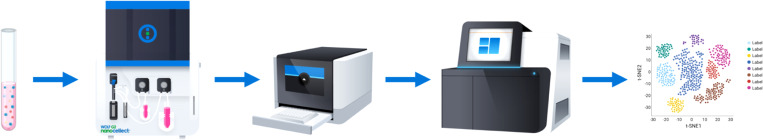
WOLF to 10x Genomics Workflow. Cells and tissues must first be prepared as single-cell suspensions. Targeted cell types are then isolated while removing dead cells and debris on WOLF or WOLF G2. Sorted cells are then partitioned by the Chromium Controller and RNA libraries are then generated according to assay used. Sequencing can then be performed on the Illumina platform and analyzed with Cell Ranger pipeline (Image generated using Biorender).

10x Genomics technology was used to determine the genomic benefits of sorting cells through the WOLF prior to RNA library construction. PBMCs were isolated from human blood donors (San Diego Blood Bank) using a density gradient and then split into two samples: (1) an unsorted sample and (2) a sample sorted on the WOLF. In the unsorted sample, 10 000 cells were prepared to a concentration of 1000 cells/*μ*l and loaded into the Chromium Controller (10x Genomics). In the WOLF sorted sample, cells were stained with propidium iodine (PI, ThermoFisher No. R37169) to identify dead cells. Sorting gates were then set to exclude debris, doublets, and PI+ dead cells. 10 000 of these sorted cells were prepared at 1000 cells/*μ*l and loaded into the Chromium Controller. Both samples were resuspended in PBS +2% FBS. RNA libraries were then generated using the Chromium Single Cell 3′ v3 Kit (10x Genomics, #No. 1000092) and sequenced on the Illumina HiSeq 4000. Sequencing analysis was performed using the Cell Ranger single-cell analysis pipeline, which includes demultiplexing, alignment, counting barcodes, and other gene expression analysis^12^ (Fig. 2).

### Single-cell RNA sequencing of iPSCs

Reprogrammed iPSCs from human skin fibroblasts (Coriell Institute, GM23338) were divided into three samples: (1) unsorted control; (2) cells sorted on a conventional high-pressure droplet sorter, FACSAria^TM^ II (Beckton-Dickenson); and (3) cells sorted on the low-pressure microfluidic WOLF Cell Sorter. Prior to sorting, propidium iodide (PI, ThermoFisher No. R37169) was used to identify dead cells. On BD FACSAria^TM^ II, cells were sorted by the Sanford Burnham Prebys Flow Core using a 100 *μ*m nozzle and pressure reduced to 20 psi. On the WOLF, PBS +0.05% BSA was used as the sheath. WOLF sort gates were set to exclude debris using BSC-H/FSC-H, and doublets with FSC-H/FSC-W. Live cells were gated as the PI-negative population. BD FACSAria^TM^ II sort gates were set to exclude debris using SSC-A/FSC-A and doublets with FSC-H/FSC-W and SSC-W/SSC-A. Live cells were also identified as the PI-negative population. 50 000 cells were sorted on both BD FACSAria^TM^ II and NanoCellect WOLF into a FACS tube precoated with 0.5 ml of PBS+ 0.05% BSA to increase cell recovery. All samples were then centrifuged and re-suspended at ∼1000 cells/*μ*l. An estimated 10 000 cells were then loaded into the 10x Chromium Controller. cDNA libraries were generated using the Chromium Next GEM Single Cell 3′ Library Construction Kit v3.1 (10x Genomics, No. 1000092) and sequenced with the Illumina NovaSeq 6000 Sequencing System (Fig. 2). All samples were analyzed with the 10x Genomics Cell Ranger pipeline to evaluate the sample and sequencing quality.

## RESULTS

### Improved library complexity and quality of PBMCs

**FIG. 3.**
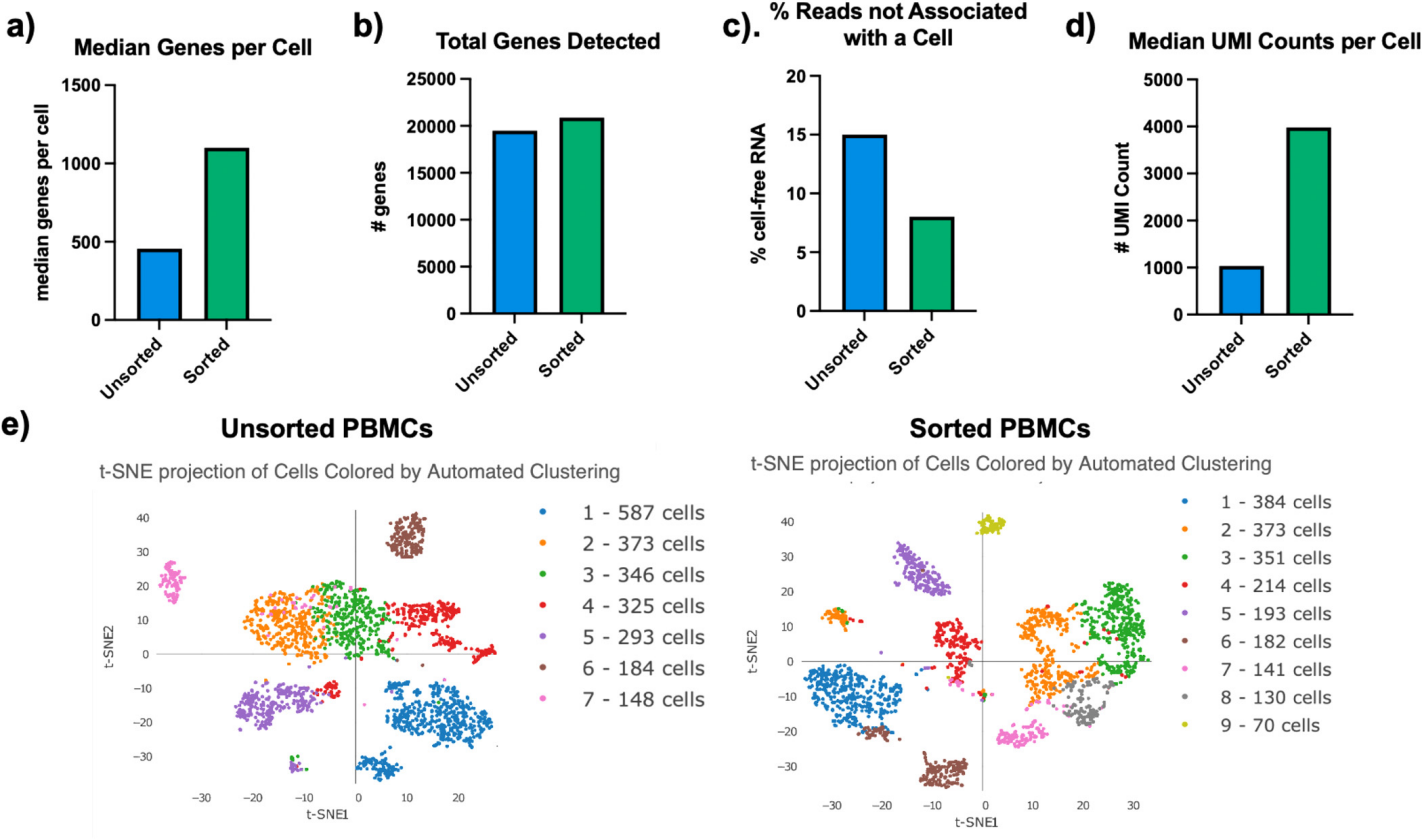
Cell Ranger summary of PBMCs. (a) Median genes per cell in the unsorted PBMCs and WOLF sorted PBMCs. (b) Total genes detected. (c) Fraction of reads that were not associated with a cell and (d) Median UMI counts per cell with a cell in the unsorted and WOLF sorted sample. (e) t-SNE projection of cell colored by automating clustering in unsorted and WOLF sorted PBMCs.

The Cell Ranger performance sequencing summary on the PBMC samples showed 2256 cells captured in the unsorted sample and 2038 cells captured in our WOLF sorted sample. There were more than twice the “median genes per cell” in our WOLF sorted sample (1101genes) compared to the unsorted sample (456 genes) [Fig. 3(a)]. In addition, there were more total genes in the WOLF sorted sample [Fig. 3(b)]. Having a higher median of genes per cell and more total genes detected both indicate higher library complexity in the WOLF sorted sample. There was also nearly twice the amount of cell-free RNA contamination in the unsorted sample, relative to the WOLF sorted sample as determined by the percentage of reads that were not associated with a cell [Fig. 3(c)]. These results highlight that there was less contamination from dead cells in the WOLF sorted sample. Furthermore, there were approximately three times more “unique molecular identifiers (UMIs) detected per cell” in our WOLF sorted sample. This reflects a higher number of RNA transcripts per cell detected in the WOLF sorted sample [Fig. 3(d)]. Furthermore, t-distributed stochastic neighbor embedding (t-SNE) projection of cells colored by automated clustering of cells that have similar transcriptional profiles produced only seven clusters of cells in the unsorted sample, relative to nine clusters of cells in the WOLF sorted sample. In addition, clusters were more distinct in the WOLF sorted sample [Fig. 3(e)]. These results further indicate that background contamination was reduced, and a higher sequencing depth was achieved in the WOLF sorted sample, which allowed for more cell types to be identified.

### Microfluidic vs droplet cell sorting of iPSCs

**FIG. 4.**
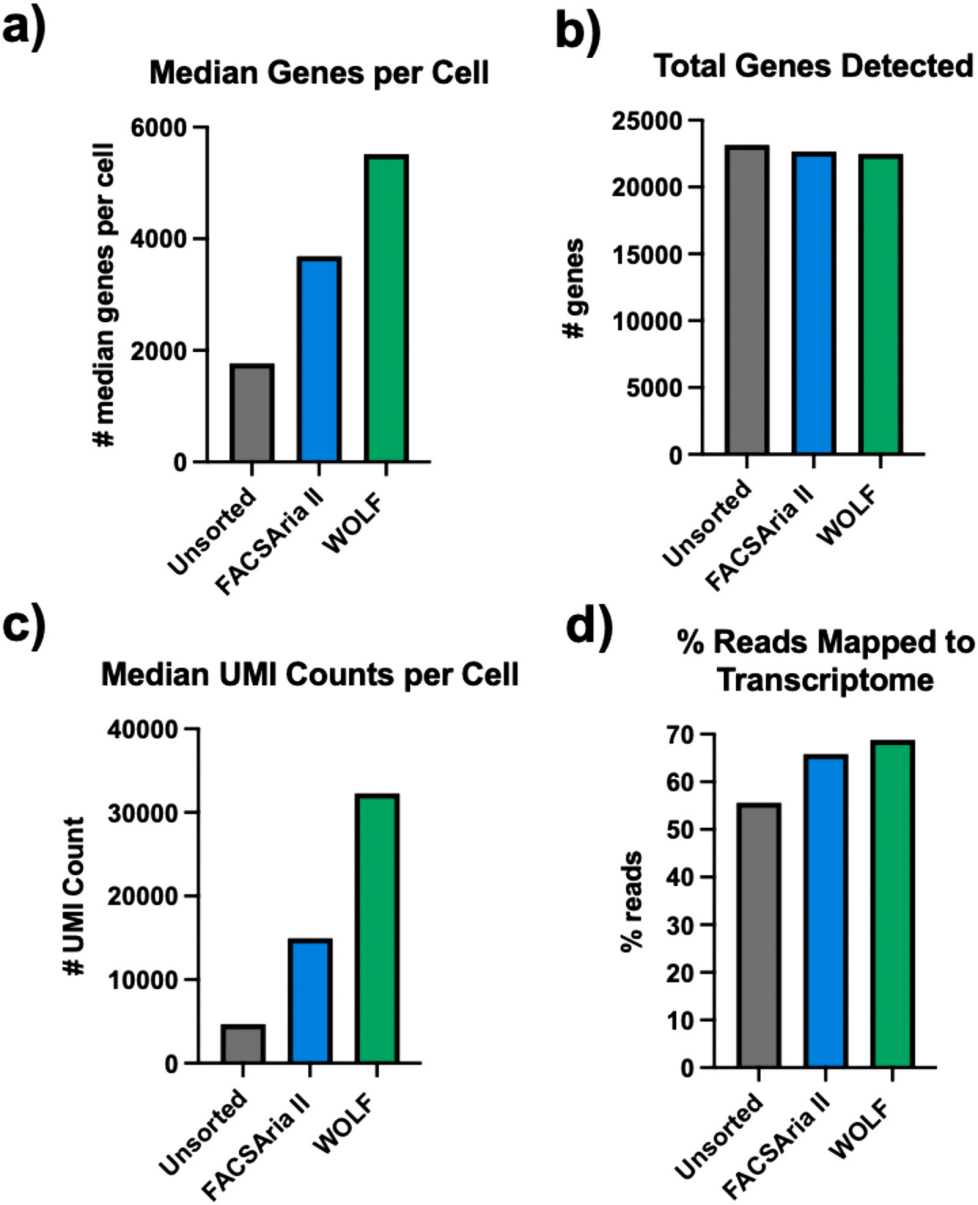
Cell Ranger summary of iPSCs. (a) Median genes per cell. (b) Total genes detected. (c) Median UMI Counts per Cell and (d) % Reads that mapped to the transcriptome in the unsorted, FACSAria II and WOLF sorted samples (n = 1).

The Cell Ranger pipeline was also performed for the iPCS samples in order to examine sample quality. The WOLF had a 3-fold increase over the unsorted cells, while the BD FACSAria II sorted sample had only 2-fold more than the unsorted cells in the “median genes per cell” [Fig. 4(a)]. No notable difference in the “total genes detected” was observed [Fig. 4(b)]. The WOLF sorted sample “median UMI counts per cell” was increased nearly 7-fold (32 239) over the unsorted population (4668) and 2-fold over the BD FACSAria II sorted population (14 949) [Fig. 4(c)]. These results reveal that a higher library complexity is achieved when debris and dead cells are removed from the sample. Finally, the “% Reads Mapped to Transcriptome” shows the percentage of the reads that uniquely map to a specific gene. A low percentage indicates the presence of fragmented RNA that cannot be unequivocally mapped. These results show an improved percentage of reads that mapped to the transcriptome in both the Aria (65.8%) and WOLF (68.8%) after sorting [Fig. 4(d)].]

## DISCUSSION

Removing debris and dead cells before doing downstream genomics assays is critical for generating high-quality sequencing results. A cleaner sample prior to library generation builds greater confidence that the results include cells that are of high quality. Two critical samples were used to demonstrate the benefits that a microfluidic cell sorter can have for genomic sample preparation. Genomics studies using PBMCs has led to many novel discoveries in cancer^13,14^ and several infectious diseases.^15,16^ In addition, single-cell RNA-sequencing on iPSCs has led to several novel findings on heterogeneity of tissue regeneration.^17,18^

In summary, these experiments demonstrate that the quality of sequencing results is directly dependent on how the sample is prepped prior to sequencing. Cell sorters serve as a valuable tool to isolate target cells while removing dead cells, debris, and doublets which is critical before performing any downstream omics application. Both sample types that were sequenced demonstrated that using a cell sorter upstream of genomic applications can result in improved library complexity, enabling more data per cell. This is a significant discovery that will help researchers to reduce reagent and sequencing costs. Furthermore, these results suggest that the use of a gentle microfluidic cell sorter may generate more favorable results compared to traditional high pressure cell sorters as observed by the higher library complexity obtained by the WOLF sample; however, future experiments with more samples would need to be conducted to provide more supporting evidence. However, the novelty here is that this microfluidic cell sorter which uses sorting conditions that are ideal for fragile cell types due to lower pressures and flexible sheath composition is another way in which scientists can remove unwanted cells and debris from their sample. In summary, these results show that microfluidic cell sorters such as the WOLF serve as a robust cell sorter to use when preparing samples for omic applications.

## Data Availability

The data that support the findings of this study are available from the corresponding author upon reasonable request.
